# The Incidence of Breast Cancer Lymph Node Involvement in Trinidad and Tobago

**DOI:** 10.7759/cureus.62406

**Published:** 2024-06-14

**Authors:** Akshay Maharaj, Kwamé Olivers, Riyad Mohammed, Levi Ramcharan, Vinash Deyalsingh, Tarini Mahase, Mickhaiel Barrow, Shaheeba Barrow, Robyn Cabral, Brittney Lutchman

**Affiliations:** 1 Internal Medicine, Port of Spain General Hospital, Port of Spain, TTO; 2 Emergency, Eric Williams Medical Sciences Complex, Tacarigua, TTO; 3 Ophthalmology, Port of Spain General Hospital, Port of Spain, TTO; 4 Pathology, Port of Spain General Hospital, Port of Spain, TTO; 5 General Surgery, Port of Spain General Hospital, Port of Spain, TTO

**Keywords:** sentinel lymph node (sln), spread, involvement, t stage, lymph node, nodal, breast cancer, trinidad, caribbean

## Abstract

Objective

This study aims to investigate breast cancer lymph node involvement in a West Indian population while correlating it with various histological parameters and evaluating the role of the sentinel lymph node biopsy.

Method

This is a retrospective study where histology reports for all breast cancer-related biopsies from 2018 to 2021, totaling 813 samples, were obtained. Histological parameters from these reports were extracted into a spreadsheet and analyzed using Statistical Product and Service Solutions (SPSS, version 28.0; IBM SPSS Statistics for Windows, Armonk, NY) software for TNM staging and axillary and sentinel lymph node dissections, among other fields found in histology reports.

Results

In 44.8% of cases, patients present at the T2 stage with associated lymph node spread. Each T stage had more lymph nodes involved than uninvolved for tumors sized T2 and higher. Inversely, for tumors staged under T2, there were generally more uninvolved lymph nodes than involved ones. Larger tumors were found to have advanced N staging, especially in the T3 category, where a significantly higher proportion of cases were found to have N2 and N3 staging compared to the other T stages. This trend is also seen in M staging, where larger tumors metastasize more than smaller tumors (40% for T4a, 0% for T1). Despite significant lymph node involvement being observed, sentinel lymph node biopsies were usually negative.

Conclusion

More patients in this population present with lymph node involvement than without. Larger breast cancer tumors are associated with greater lymph node involvement, particularly at T2 and higher stages. Sentinel lymph node biopsies can be omitted in smaller breast cancer tumors up to 2 cm in size, and the local recurrence rate is low despite a false-negative rate of around 10% in upfront sentinel lymph node biopsy.

## Introduction

Breast cancer imposes a great burden, and staging this cancer is essential in guiding management. Tumor size and lymph node involvement are closely linked to breast cancer. Larger tumors are more likely to spread to nearby lymph nodes, and lymph node involvement strongly predicts a worse prognosis and an increased risk of recurrence [1,2]. Several studies have investigated the relationship between tumor size and lymph node status, with varying results.

Sopik et al. conducted a study of 819,647 patients with invasive breast cancer and found a strong correlation between tumor size and lymph node involvement [3]. The study discovered that the likelihood of lymph node involvement increased with tumor size, with lymph node involvement increasing from 10 mm to 60 mm tumors. In contrast, Andea et al. found that a larger tumor diameter correlates with greater lymph node involvement in unifocal invasive breast cancers. However, in invasive breast tumors with multiple nodules, it was unclear whether tumor size correlates with lymph node dissemination [4].

Ahmad et al. determined that of 120 breast cancer tumors, they were mainly T2 or T3, and 80 of those cases involved the axillary lymph nodes. Over 90% of these cases were grade 2 tumors [5]. The literature mentions other factors that affect lymph node spread. For instance, hormone-positive or HER-negative receptor tumors were found to have more lymph node invasion than triple-negative tumors [6]. Isheden et al., on the other hand, discovered that hormone-negative tumors spread 1.25 times faster than hormone-positive tumors [7].

Despite the clear relationship between tumor size and lymph node involvement, there is an ongoing debate about the method of lymph node sampling. A meta-analysis after neoadjuvant chemotherapy of 3,578 participants from 27 trials determined that sentinel node biopsies had a false negative rate of 15% and, thus, a sensitivity of 85% [8]. Similarly, other studies have found sentinel node biopsies to be an unacceptable standard, as their false negative rate of 12.6-14.2% is above the minimum acceptable rate of 10% [9]. This study aims to elucidate this discrepancy by assessing the role of the sentinel lymph node biopsy in terms of its sensitivity and practicality in contemporary surgery. This study also focuses on the T and N staging components and their correlation in a West Indian population. While several studies have investigated the relationship between T and N staging in breast cancer, the precise nature of this association remains unclear in this population.

## Materials and methods

Institutional Review Board approval was obtained from the University of The West Indies Campus Ethics Committee (Ref: CREC-SA.2595/03/2024) for the commencement of this retrospective study. This study took place at Port of Spain General Hospital, Trinidad and Tobago. The histology department's medical record system was accessed. The system was searched for the phrase “breast cancer.” A list of all histology reports for breast cancer biopsies was generated. All histology reports from January 1, 2018, to July 12, 2021, were printed. This generated a sample size of 813 female patients. Each report was assigned a unique identifier. Various fields of data were extracted from the reports including patient age, ethnicity, clinical presentation, procedures performed, staging, size of nodal metastases, histology type, invasion of margins, subtype, tumor grade, differentiation, presence of lymphovascular invasion, extranodal extension, Allred score, Ki-67 level, receptor status, number of sentinel lymph nodes biopsied, number of axillary lymph nodes involved, and if neoadjuvant therapy was done. The number of axillary lymph nodes was categorized and recorded as either less than five nodes, 5-10 nodes, or more than 10 nodes.

These fields were assigned a numerical code for each possibility. For instance, African patients were assigned the number “1,” and Indian patients were assigned the number “2.” These numerical codes were entered and tabulated into a spreadsheet. The spreadsheet was then analyzed using Statistical Product and Service Solutions (SPSS, version 28.0; IBM SPSS Statistics for Windows, Armonk, NY). The sample was split into groups depending on the N stage (N0 vs N1 vs N2 vs N3). SPSS was utilized to calculate mean age and standard deviation along with frequency counts and percentages for each group separately. The whole sample was then split according to the number of axillary lymph nodes involved (<5 nodes, 5-10 nodes,>10 nodes) and according to the T staging (T1, T2, T3, T4). SPSS was again utilized to calculate mean age and standard deviation along with frequency counts and percentages for each of these splits. A chi-square analysis was also done to determine the association between T and N staging. These results were inferred against a background of relevant research done both regionally and internationally.

## Results

**Figure 1:**
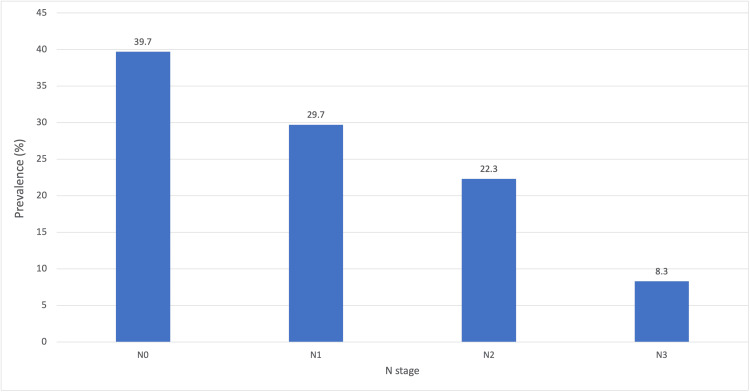
Showing the Prevalence of Each N Stage

This study enrolled 813 cases of diagnosed breast cancer patients. Of the 813 cases, only 33.3% (n=271) were T staged, 28.1% (n=228) were N staged, and 22.7% (n=185) were M staged on histology reports. T staging was found to be T1 (13.6%, n=37), T2 (41.7%, n=128), T3 (24.7%, n=67), and T4 (6.2%, n=17). N staging was found to be N0 (39.7%, n=91), N1 (29.7%, n=68), N2 (22.3%, n=51), and N3(8.3%, n=19). M stage was found to be Mx (56.2%, n=104), M0 (16.8%, n=31), and M1 (26.5%, n=49). The distribution of cases and parameters across each N stage are represented in Figure 1 and Table 1. 

**Table 1:** Comparing Various Parameters for Each N Stage Others are a collective term for minor subtypes that were grouped together. DCIS: ductal carcinoma in situ; HER2: human epidermal growth factor 2; IDC: invasive ductal carcinoma; ILC: infiltrating lobular carcinoma

Variable	All N Stages	N0 Stage	N1 Stage	N2 Stage	N3 Stage
Number of cases	299	39.7% (n=91)	29.7% (n=68)	22.3% (n=51)	8.3% (n=19)
Mean age of patients (years)	56.4	58.67	54.87	55.55	54.26
Most common receptor types	Hormone receptor-positive/HER negative (18.3%, n=32)	Hormone receptor-positive/HER negative (20.9%, n=19)	Hormone receptor positive/HER negative and triple negative (each at 14.7%, n=10)	Hormone receptor positive/HER negative (19.6%, n=10)	Triple negative (21.1%, n=4), followed by hormone positive/HER negative (15.8%, n=3)
Most common clinical presentation	Mass (45.9%, n=105)	Mass (46.2%, n=42)	Mass (50%, n=34)	Mass (37.3%, n=19)	Mass (52.6%, n=10)
Most common procedure performed	Mastectomy with axillary clearance (43.7%, n=100)	Mastectomy with axillary clearance (42.9%, n=39)	Mastectomy with axillary clearance (42.6%, n=29)	Mastectomy with wide axillary clearance (47.1%, n=24)	Mastectomy with wide axillary clearance (42.1%, n=8)
Most common T stages	T2 (38.9%, n=89)	T2 (42.9%, n=39)	T2 (45.6%, n=31)	T2 (31.4%, n=16)	T2 (15.8%, n=3)
	T3 (27.1%, n=62)	T3 (14.3%, n=13)	T3(22.1%, n=15)	T3 (47.1%, n=24)	T3 (52.6%, n=10)
Metastasis	Unmentioned (36.7%, n=84)	Unmentioned (37.4%, n=34)	Unmentioned (39.7%, n=27)	Unmentioned (31.4%, n=16),	Unmentioned (36.8% n=7)
	Mx (38.9%, n=89)	Mx (42.9%, n=39)	Mx (36.8%, n=25)	Mx (35.3%, n=18)	Mx (36.8%, n=7)
	M1 (15.7%, n=36)	M1 (4.4%, n=4)	M1 (19.1%, n=13)	M1 (27.5%, n=14)	M1 (26.3%, n=5)
	M0 (8.7%, n=20)	M0 (15.4%, n=14)	M0 (4.4%, n=3)	M0 (5.9%, n=3)	M0 (0%)
Nodes involved in sentinel lymph node biopsy	0 nodes (93.9%, n=215)	0 nodes (87.9%, n=80)	0 (97.1%, n=66)	0 nodes (98%, n=50)	0 nodes (100%, n=19)
	1 node (4.8%, n=11)	1 node (8.8%, n=8)	1 node (2.9%, n= 2)	1 node (2%, n=1)	____
	2 nodes (1.3%, n=3)	2 nodes (3.3%, n=3)	___	___	___
Nodes involved in axillary node dissection	<5 nodes (59.8%, n=137)	<5 nodes (61.5%, n=56)	<5 nodes (73.5%, n=50)	<5 nodes (45.1%, n=23)	<5 nodes (42.1%, n=8)
	5-10 nodes 13.1% (n=30)	5-10 nodes (12.1%, n=11)	5-10 nodes (10.3%, n=7)	5-10 nodes (21.6%, n=11).	5-10 nodes (5.3%, n=1)
	>10 nodes (26.6%, n=61)	>10 nodes (25.3%, n=23)	>10 nodes (16.2%, n=11)	>10 nodes (33.3%, n=17)	>10 nodes (52.6%, n=10)
Size of nodal metastases	Mostly macrometastases (13.5%, n=31).	Unmentioned (94.5%, n=86)	Unmentioned (82.4%, n=56)	Unmentioned (70.6%, n=36)	Unmentioned (78.9%, n=15)
	____	Micrometastases (1.1%, n=1)	Micrometastases 4.4% (n=3)	____	____
	____	Macrometastases (4.4%, n=4)	Macrometastases (11.8%, n=8)	Macrometastases (29.4%, n=15)	Macrometastases (21.1%, n=4)
Extranodal extension	Unmentioned (87.7%, n=201)	Unmentioned (98.9%, n=90)	Unmentioned (85.3%, n=58)	Unmentioned (76.5%, n=39)	Unmentioned (73.7%, n=14)
	Present (12.2 %, n=28)	Present (1.1%, n=1)	Present in 14.7% (n=10)	Present (21.6%, n=11) and <2 mm (2%, n=1)	Present (26.3%, n=5)
Most common histology	IDC (45%, n=103), followed by IDC/DCIS (19.2%, n=44)	IDC (36.3%, n=33), followed by IDC/DCIS (16.5%, n=15) and invasive lobular carcinoma (13.2%, n=12)	IDC (44.1%, n=30), followed by IDC/DCIS (27.9%, n=19) and invasive lobular carcinoma (4.4%, n=3)	IDC (56.9%, n=29), followed by ILC only (19.6%, n=10) and IDC/DCIS (13.7%, n=7).	IDC only (57.9%, n=11), followed by ILC only (21.1%, n=4) and IDC/DCIS (15.8%, n=3).
Tumor margins involvement	16.6% (n=38)	12.1% (n=11)	16.2% (n=11)	19.6% (n=10)	31.6% (n=6)
Most common subtype	Adenocarcinoma (10.9%, n=25), followed by comedo (10.5%, n=24).	Adenocarcinoma 12.1%(n=11), followed by comedo in 8.8% (n=8)	Comedo (16.2%, n=11), followed by adenocarcinoma (8.8%, n=6).	Other (43.1%, n=22), followed by adenocarcinoma (13.7%).	Other (47.4%, n=9), followed by comedo (15.8%)
Grade	Low grade (4.8%, n=11)	Low grade (7.7%, n=7)	Low grade (2.9%, n=2)	Low grade (3.9%, n=2)	Low grade (0%)
	Intermediate grade (49.3%, n=113)	Intermediate grade (51.6%, n=47)	Intermediate grade (48.5%, n=33)	Intermediate grade (56.9%, n=29)	Intermediate grade (21.1%, n=4)
	High grade (34.9%, n=80)	High grade (27.5%, n=25)	High grade (41.2%, n=28)	High grade (27.5%, n=14)	High grade (68.4%, n=13)
Differentiation	2.2% (n=5) poorly differentiated	3.3% (n=3) poorly differentiated	11.8% (n=8) poorly differentiated	3.9% (n=2) poorly differentiated	10.5% (n=2) poorly differentiated
	3.1% (n=7) moderately differentiated	6.6% (n=6) moderately differentiated	1.5% (n=1) moderately differentiated	0% moderately differentiated	0% moderately differentiated
	6.6% (n=15) well differentiated	4.4% (n=4) well differentiated	1.5% (n=1) well differentiated	0% well differentiated	0% well differentiated
Lymphovascular invasion	Identified (29.7%, n=68)	Identified (11%, n=10)	Identified (39.7%, n=27)	Identified (43.1%, n=22)	Identified (47.4%, n=9)
	Suspected (9.2%, n=21)	Suspected (7.7%, n=7)	Suspected (13.2%, n=9)	Suspected (7.8%, (n=4)	Suspected (5.3%, n=1)
Ki-67 expression	<6% intensity in 0.4% (n=1)	<6% intensity in 0%	No expression	<6% intensity in 2% (n=1)	No expression
	6-10% intensity in 0.4% (n=1)	6-10% intensity in 1.1% (n=1)		6-10% intensity in 0%	
	>10% intensity in 3.9% (n=9)	>10% intensity in 7.7% (n=7)		>10% intensity in 3.9% (n=2)	
Neoadjuvant therapy given	8.3% (n=19)	5.5% (n=5)	10.3% (n=7)	13.7% (n=7)	No recorded cases

Regarding tumor grade, it was found that grade 3 tumors (n=207) exhibited a more advanced N staging (N3: 6.3%, n=13) compared to grade 1 tumors (n=42), which had 0% N3 staging. Additionally, the N2 staging was higher in grade 3 tumors (6.8%, n=14) compared to grade 1 tumors (4.8%, n=2). Grade 3 tumors were also larger, with 19.8% (n=41) being classified as T2 and 15% (n=31) as T3, whereas grade 1 tumors had 16.7% (n=7) classified as T2 and only 2.4% (n=1) as T3. One study showed that 25% of grade I tumors were associated with lymph node metastases, compared to 52.3% of grade III cancers. The size of the tumor was also a significant effect, with positivity ranging from 0% for T1a tumors to 88.9% for T3 malignancies [10].

N3 was found to be most frequently associated with hormone-positive (estrogen receptor/progesterone receptor (ER/PR) positive)/HER2-negative (19.6% of cases). N1 staging was also found to have a similar proportion of hormone-positive/HER2-negative cases (20.9%, n=19). In the N1 cases, 11% (n=10) showed lymphovascular invasion, whereas 43.1% showed lymphovascular invasion in the N3 group.

Table 2 illustrates the analysis of data related to axillary lymph node dissection in terms of the number of lymph nodes involved. In the group with fewer than five nodes, 84% (n=684) of cases were observed, whereas 5.9% (n=48) had five to 10 nodes removed, and 9.9% (n=81) had more than 10 nodes removed. The mean age of the patients was 55.7 years, with a standard deviation of 14.5 for those with fewer than five nodes removed; 57.0 years, with a standard deviation of 11.9 for those with five to 10 nodes removed; and 54.9 years, with a standard deviation of 13.6 for those with more than 10 nodes removed. The age ranges for these groups were 97, 55, and 83, respectively. The most common T stages were T2 (10.8%, n=78) and T3 (6.6%, n=45) for patients with less than five nodes removed, T2 (20.8%, n=10) and T3 (18.8%, n=9) for those with five to 10 nodes removed and T2 (35.8%, n=29) and T3 (14.8%, n=12) for those with more than 10 nodes removed. The percentage of patients in the M1 stage was 5.1% (n=35) for less than five nodes involved, 10.4% (n=5) for five to 10 node involvement, and 11.1% (n=9) for greater than 10 nodes involvement on axillary lymph node dissection.

**Table 2:** Distribution of Tumor Sizes and Sentinel Nodes Across Each N Stage ≤ means less than equal to, therefore, a tumor at 0.2 cm classifies as T1c

Tumor Size	N0	N1	N2	N3
T1 (<2 cm)	46.7%	26.7%	6.7%	0%
T1mi (<0.1 cm)	0%	50%	0%	0%
T1a (>0.1 cm but ≤0.5 cm)	75%	25%	0%	0%
T1b (>0.5 cm but ≤10 mm)	9.1%	9.1%	0%	18.2%
T1c (>.0.1 cm but ≤0.2 cm)	45.5%	22.7%	0%	4.5%
T2 (>0.2 cm but ≤ 0.5 cm)	34.5%	27.4%	14.2%	2.7%
T3 (>0.5 cm)	19.4%	22.4%	35.8%	14.9%
T4a	40%	20%	0%	0%
T4b	16.7%	0%	41.7%	0%
Nodes involved in sentinel node biopsy at each N stage	0 nodes (87.9% of all N0 cases)	0 nodes (97.1% of all N1 cases)	0 nodes (98% of all N2 cases)	0 nodes (100% of all N3 cases)
	1 node (8.8% of all N0 cases)	1 nodes(2.9% of all N1 cases)	1 nodes (2% of all N2 cases)	-
	2 nodes (3.3% of all N0 cases)	-	-	-

Chi-square statistics were used to examine the association between the categorical variables (T stage and N stage). There was a significant association at a 5% significance level between the T stage and the N stage (x2 = 677.322 df=44; P value=0.000).

As regards T1mi tumors, Nx and N1 presentations each represented 50% (n=1). Unmentioned metastasis and Mx each accounted for 50% (n=1). The histologic variant ductal carcinoma in situ (DCIS) only accounted for 50% (n=1), while the “other” variants accounted for 50% (n=1). All cases (100%, n=2) of T1mi tumors had zero lymph node involvement of sentinel lymph node biopsy. Axillary lymph node dissection involved less than five nodes in 50% (n=1) of cases and greater than 10 nodes in 50% (n=1) of cases. The size of nodal metastasis was unmentioned for 50% (n=1) of cases, while micrometastasis was present in 50% (n=1) of cases. The extranodal extension was unmentioned for all (100%, n=2) cases.

For T1a tumors, N0 and N1 presentation accounted for 75% (n=3) and 25% (n=1), respectively. Unmentioned metastasis represented 25% (n=1), while Mx represented 75% (n=3). Invasive ductal carcinoma (IDC) only, IDC and DCIS, and DCIS only each represented 25% (n=1). All (100%, n=4) cases had zero lymph node involvement on sentinel lymph node biopsy. In contrast, less than five nodes (75%, n=3) were primarily involved in axillary lymph node dissection, followed by five to 10 nodes (25%, n=1). The size of nodal metastases and extranodal extension were unmentioned for all cases (100%, n=4).

Concerning T1b tumors, Nx (63.6%, n=7) was the most common N-stage presentation, followed by N3 (18.2%, n=2), with N0 and N1 each representing 9.1% (n=1). Unmentioned metastasis was 81.8% (n=9), while M1 and Mx each represented 9.1%(n=1). The most common histologic variant was IDC (45.5%, n=5), followed by IDC and DCIS (36.4%, n=4) and Paget’s/ DCIS/infiltrating lobular carcinoma (ILC) (9.1%, n-=1). “Other” variants accounted for 9.1%(n=1) of cases. All cases had zero lymph node involvement (100%, n=11). All (100%, n=11) cases had less than five nodes involved in axillary lymph node dissection. The size of nodal metastases and extranodal extension was unmentioned in 100% (n=11) of cases.

With respect to T1c tumors, N0 (45.55%, n=10) was the most common N-stage presentation, followed by Nx (27.3%, n=6), N1(22.7%, n=5), and N3 (4.5%, n=1). Unmentioned metastasis accounted for 59.1% (n=13), while Mx and M1 represented 36.4% (8) and 4.5% (n=1), respectively. IDC only and IDC and DCIS each accounted for 36.4% (n=8) of histologic variants, while “other” variants and ILC accounted for 13.6% (n=3) and 9.1% (n=2), respectively. The number of nodes involved in sentinel lymph node biopsy was predominantly zero (77.3%, n=17), followed by one (22.7%, n =5). Less than five nodes (72.7%, n=16) were primarily involved in axillary lymph node dissection, followed by five to 10 nodes (18.2%, n=4) and greater than 10 nodes (9.1%, n=2). The size of nodal metastasis was unmentioned in 77.3% (n=17) of cases, while macrometastases were present in 18.2% (n=4) of cases. The extranodal extension was unmentioned in all cases (100%, n=22).

For T2 tumors, N0 (34.5%, n=39) was the most prevalent N-stage presentation, followed by N1, Nx, N2, and N3 at 27.4% (n=31), 21.2% (n=24), 14.2% (n=16), and 2.7% (n=3), respectively. Unmentioned metastasis accounted for 45.1% (n=51) followed, while Mx, M1, and M0 represented 44.2% (n=50), 7.1% (n=8), and 3.5% (n=4), respectively. The histologic variant most commonly present was IDC only (38.9%, n=44), followed by IDC and DCIS (22.1%, n=25), then by “other” at 19.5% (n=22), and ILC only (17.7%, n=20). ILC and DCIS and DCIS only each accounted for less than 1%. The number of nodes involved in sentinel lymph node biopsy was primarily zero (94.7%, n=107), followed by one (3.5%, n=4) and two (1.8%, n=2). Less than five nodes (65.5%, n=74) were primarily involved in axillary lymph node dissection, followed by greater than 10 nodes (25.7%, n=29) then five to 10 nodes (8.8%, n=10). The size of nodal metastases that were unmentioned represented 91.2% (n=103), while macrometastases accounted for 8.8% (n=10). Extranodal extension was unmentioned in 89.4% (n=101) of cases but present in 10.6% (n=12) of cases.

With respect to T3 tumors, N2 (35.8%, n=24) was the most prevalent N-stage presentation, followed by N1, N0, N3, and Nx at 22.4% (n=15), 19.4% (n=13), 14.9 % (n=10), and 7.5% (n=5), respectively. Unmentioned metastasis, Mx and M1 each accounted for 31.3% (n=21) each, and M0 represented 6%. The histologic variant most commonly present was IDC only (56.7%, n=38) followed by ILC only (16.4%, n=11), then IDC and DCIS (14.9%, n=10), “other” at 7.5% (n=5) and DCIS only (1.5%, n=1). The number of nodes involved in sentinel lymph node biopsy was primarily zero (95.5%, n=64), followed by one (3%, n=2) and two (1.5%, n=1). Less than five nodes (67.2%, n=45) were primarily involved in axillary lymph node dissection, followed by greater than 10 nodes (17.9%, n=12), then five to 10 nodes (13.4%, n=9) and five nodes (1.5%, n=1). The size of nodal metastases that were unmentioned represented 77.6% (n=52), while micrometastases and macrometastases accounted for 3% (n=2) and 17.9% (n=12), respectively. Extranodal extension was unmentioned in 80.6% (n=54) of cases but present in 19.4% (n=13) of cases.

Regarding T4a tumors, Nx and N0 presentations each accounted for 40% (n=2) of N-stage presentations, followed by N1 (20%, n=1). Unmentioned metastasis and M1 each accounted for 40% (n=2), followed by Mx at 20% (n=1). IDC only histology represented all (100%, n=5) of cases. All cases (100%, n=5) had 0 lymph node involvement on sentinel lymph node biopsy. Similarly, all cases (100%, n=5) had <5 nodes involved in axillary lymph node dissection. The size of nodal metastases was unmentioned in 80% (n=4) of cases, but macrometastases accounted for 20% (n=1). The extranodal extension was unmentioned for all (100%, n=5) cases.

For T4b tumors, Nx and N2 presentations each accounted for 41.7% (n=5) of N-stage presentations, followed by N0 (16.7%, n=2). Unmentioned metastasis accounted for 41.7% (n=5), followed by Mx at 58.3% (n=7). IDC only accounted for 66.7%(n=8) of cases, followed by “other” at 25% (n=3) and IDC and DCIS at 8.3% (n=1). All cases (100%, n=12) had 0 lymph node involvement on sentinel lymph node biopsy. Alternatively, <5 nodes (66.7%, n=8) were mostly involved in axillary lymph node dissection, followed by greater than 10 nodes (25%, n=3) and five to 10 nodes (8.3%, n=1). The size of nodal metastases was unmentioned in 91.7% (n=11) of cases, but macrometastases accounted for 8.3% (n=1). Extranodal extension was unmentioned for all (100%, n=12) cases.

## Discussion

The study revealed more patients present with lymph node involvement (60.3%, n=138) than without (39.7%, n=91). This is likely because of screening. The most common stage they presented was N1 (29.7%, n=68) followed by N2 (22.3%, n=51). With respect to the axillary lymph node dissection, the majority presented with less than five nodes involved. An increase in lymph node involvement is associated with an increase in tumor size, specifically a stronger association with T3 tumors. As N staging increases, so does the likelihood of the tumor invading the margins as well as the size of nodal metastases. IDC remains the dominant histology among all the N stages. There were no significant differences in the mean ages at presentation among the N stages.

Based on the results obtained, it can be seen that the majority of patients present at T2, followed by T3, whereas a minority present at T1 and T4. A considerable number of these patients with T2-sized tumors were associated with lymph node spread in 44.8% of cases (N1 stage in 27.4%, n=31; N2 14.2%, n=16; N3 2.7%, n=3) but also had no lymph node involvement in 34.5% of cases. Tumors sized T2 had a greater propensity to metastasize (7.1%, n=8) than to not (3.5%, n=4).

This study revealed a clear correlation between tumor size and larger tumors that were found to have advanced N staging. This is seen particularly in the T3 category where a significantly higher proportion of cases were found to have N2 and N3 staging compared to the other T stages. Furthermore, this is bolstered by the T4b group of tumors having the highest percent of cases staged at N2 (41.7%, n=5) compared to the other T stages. This trend is also seen in M staging, where larger tumors metastasize more than smaller tumors (T4a 40%, n=2, T1 0%). As expected, the size of these nodal metastases was found to be larger in more advanced T stages. A study that examined the correlation between lymph node status and tumor growth determined that 26 out of 50 (52%) patients classified as N1; T2, T3, and T4 showed an increase in lymph node positivity, as three out of five (60%) T3 patients had N2 lymph node status, and all T4 patients had N3 lymph node status [11]. Legha et al. discovered that the average number of lymph nodes that are clinically and histopathologically positive for metastasis increases as the average size of the breast tumor increases [12].

From tumors sized T2 and higher, each T stage had more lymph nodes involved than uninvolved. Inversely, tumors staged under T2, generally had more uninvolved lymph nodes than involved ones. Therefore, it can be inferred that for tumors sized T2 and above, the odds of lymph node involvement become significantly higher. This is consistent with a previous study in 2019, which determined that N2/N3 lymph node metastases occur particularly with T2 tumors [13].

Albeit patients most commonly presented with a mass in the vast majority of cases, it should also be noted that a small percentage of them present with enlarged lymph nodes in the T2 and T3 groups (2.7 and 1.5%, respectively), whereas smaller tumors presented with 0% enlarged lymph nodes.

Similar findings have been reported in other studies; one hospital found 31 patients presented with axillary lymphadenopathy over a 25-year span [14]. Additionally, it can be said that high-grade tumors are associated with larger sizes and more lymphovascular invasion and, thus, a higher N stage compared to low-/intermediate-grade tumors.

Despite a significant lymph node involvement in this population and the confirmation that higher N staging corresponds to more axillary lymph nodes involved in dissections, the sentinel lymph node biopsies were largely unremarkable. The sentinel biopsies were mostly negative (0 nodes involved) even in larger tumors that had more lymph node involvement. For example, T1, T1mi, T1a, T1b, T4a, and T4b all had 0 sentinel nodes in 100% of cases. Meanwhile, 94.7% (n=107) and 95.5 %(n=64) of sentinel lymph nodes were negative in T2 and T3, respectively. This could potentially be because of bias from the low number of cases in T4a and T4b tumors. Alternatively, as N staging becomes more advanced, the number of sentinel nodes yields falls. This is seen in Table 2, where N3 had 0 sentinel nodes in 100% (n=19) of cases, while N0 had positive nodes in 12.1% of cases. This begs the question of how reliable sentinel lymph node biopsies are in advanced lymph node spread. This study has referenced other literature showing sentinel node biopsies to have false negative rates of 15% and 12.6%-14.2% [8,9]. This corresponds to T1a-sized tumors in this population, as it had the highest association with N0 (75%, n=3). A study done on only T1-2 N0 tumors determined that sentinel lymph node biopsies had a 97.2% predictive rate of axillary involvement [15]. Meanwhile, there are several studies comparing sentinel lymph node biopsy to axillary lymph node dissection, which all showed that axillary dissection was associated with more morbidity and no gain in survival [16-19].

The SOUND trial concluded that the omission of axillary surgery was noninferior to sentinel lymph node biopsy in patients with breast cancer up to 2 cm and a negative result on ultrasonography of the axillary lymph nodes [20]. Hence, there is evidence for abandoning sentinel lymph node biopsy in smaller tumors. There are data showing that the local recurrence rate is low despite a false negative rate of around 10% in upfront sentinel lymph node biopsy [16].

While this study provides valuable insights into the landscape of breast cancer nodal spread in the Caribbean, it is essential to acknowledge certain limitations that may impact the generalizability and robustness of our findings. Firstly, the sample size in this study was relatively modest, which may limit the extrapolation of results to the entire Caribbean population. This is likely the reason for the N3 results in Table 2 (18.2% of N3 tumors being T1b and 4.5% of N3 tumors being T1c). Additionally, the study primarily focused on a specific time frame, and temporal variations in nodal involvement were not extensively explored. The diversity within the Caribbean population was considered to some extent, but the complexity of these interactions may require further investigation. Furthermore, the retrospective nature of the data collection process introduces inherent biases and limitations, such as incomplete medical records. There may exist a reporting bias in that histological features for a large portion of biopsies were not reported. Future research endeavors should aim to address these limitations, employing larger sample sizes, prospective study designs, and more comprehensive analyses to enhance the accuracy and applicability of our findings.

## Conclusions

More patients in this population present with lymph node involvement than without. Larger breast cancer tumors are associated with greater lymph node involvement, particularly at T2 and higher stages. Greater spread is also associated with higher-grade tumors. Despite significant lymph node invasion, the sentinel lymph node biopsies were usually negative, especially at higher N stages. Hence, the relevance of continuing sentinel lymph node biopsies needs to be reconsidered in the management of breast cancer. There is evidence that sentinel lymph node biopsies can be omitted in smaller breast cancer tumors up to 2 cm in size and that the local recurrence rate is low despite a false negative rate of around 10% in upfront sentinel lymph node biopsies.
